# Can participatory processes lead to changes in the configuration of local mental health networks? A social network analysis

**DOI:** 10.3389/fpubh.2023.1282662

**Published:** 2023-11-08

**Authors:** Salvador Camacho, Adriane Martin Hilber, Laura Ospina-Pinillos, Mónica Sánchez-Nítola, Débora L. Shambo-Rodríguez, Grace Yeeun Lee, Jo-An Occhipinti

**Affiliations:** ^1^Swiss Centre for International Health, Swiss Tropical and Public Health Institute, Basel, Switzerland; ^2^University of Basel, Basel, Switzerland; ^3^Department of Psychiatry and Mental Health, School of Medicine, Pontificia Universidad Javeriana, Bogota, Colombia; ^4^Brain and Mind Centre, The University of Sydney, Sydney, NSW, Australia; ^5^Computer Simulation and Advanced Research Technologies, Sydney, NSW, Australia

**Keywords:** adolescents, young people, mental health, social network analysis, system dynamics modeling, participatory systems modeling, health system, Colombia

## Abstract

Systems modeling offers a valuable tool to support strategic decision-making for complex problems because it considers the causal inter-relationships that drive population health outcomes. This tool can be used to simulate policies and initiatives to determine which combinations are likely to deliver the greatest impacts and returns on investment. Systems modeling benefits from participatory approaches where a multidisciplinary stakeholder group actively engages in mapping and contextualizing causal mechanisms driving complex system behaviors. Such approaches can have significant advantages, including that they may improve connection and coordination of the network of stakeholders operating across the system; however, these are often observed in practice as colloquial anecdotes and seldom formally assessed. We used a basic social network analysis to explore the impact on the configuration of the network of mental health providers, decision-makers, and other stakeholders in Bogota, Colombia active in a series of three workshops throughout 2021 and 2022. Overall, our analysis suggests that the participatory process of the systems dynamics exercise impacts the social network’s structure, relationships, and dynamics.

## 1. Introduction

The use of systems models offers a valuable instrument to support strategic decision-making for complex problems. A system model is a tool that can be used to understand the complex causal inter-relationships that drive population mental health outcomes and can simulate policies and initiatives (individually or in combination) to determine which are likely to deliver the best outcomes and returns on investment in youth mental health (1). Systems modeling benefits from participatory approaches where a group of stakeholders with diverse areas of expertise working across the system of interest, actively engages in mapping and contextualizing causal mechanisms driving complex system behaviors (2). This process is also referred to as participatory systems modeling (PSM). In the case of Bogota, Colombia, the result of the PSM was a computerized dynamic model of the local mental health system.

Involvement of stakeholders in the model-building process is particularly important in mental health, as it offers the opportunity to obtain the diverse perspectives of those working in and/or trying to navigate the system, including young people with lived (or living) experience of mental illness. This brings care providers and care receivers together into a joint process of learning and problem solving to strengthen the youth mental health system. Low-and middle-income countries (LMICs) have wider gaps in the adoption of evidence-based information to inform decision-making (3). Despite the uptake of decision support tools in high income countries, LMICs still struggle to implement and use these tools to inform policy (4). The reasons are multiple and complex; certainly the availability of local systems modeling expertise, and the perceived lack of quantity and quality of data act as barriers to uptake of these tools. Other possible explanations include that decision makers may not yet be familiarized with these tools, may not trust them, or their use is not part of their routine practices, and most importantly, they may not understand their value (3, 5). Hence, the participatory process of coming together with a broad range of stakeholders working across the youth mental health system provides a platform for joint questioning, learning, and trust building that may deliver positive benefits for the structure and coherence of the social network.

This process is reported to have several benefits such as improved model credibility, utility, and a robust basis for policy and planning dialogues which can lead to organized, evidence-informed decision making, and active advocacy (6). However, the impact of participatory process is commonly focused on participant engagement, their value obtained from the exercise as well as the products resulting from the process (2, 7–9). Even though some frameworks look at partnerships as a result of the interaction inside the PSM (8), these frameworks do not consider the impact of the partnerships into the whole network. Therefore, there is a paucity of evidence of the impact of PSM on professional networks, e.g., generating more connections, coordination and communication across the actors that form the network that we aim to cover with a Social Network Analysis (SNA). SNA provides a method for measuring changes in the network before and after participation in the collaborative model building process.

Colombia is an upper middle-income country with 48 million inhabitants. It has also been recognized as the most decentralized country in Latin America (10). Its health system is complex where multiple actors play diverse roles and their degree of influence over public policy also varies. For example, the Ministry of Health and Social Protection acts at a central level to create policy, but the Local Health Departments have the autonomy to enact the policy and to implement programs according to the local needs. Health system funding also has an interplay of multiple sectors (11) such as the subsidized (public), contributive (private), and special regimes (e.g., army or police) where insurers (“Entidades Administradoras de Planes de Beneficios de Salud, EAPB in Spanish) and health provider institutions are the main actors. Academia, scientific societies, non-governmental organizations (NGOs) and the civilian society to a lesser extent also contribute to influence policy.

As Koon et al. (3) postulated, the likelihood of novel information to inform public policy relies on the institution/organization’s reputation, capacity, quality and quantity of connections to decision-makers and others. Considering this, the present study aimed to detect changes in the social network as a result of the participatory process, which could result in positive outcomes such as facilitating the dialogue between stakeholders and consequently knowledge exchange and improved system coherence. This SNA also allowed us to explore the perceived roles of the stakeholders within a youth mental health professional network in Bogota, Colombia, e.g., to identify which actor is the most influential or which one has the most connections.

## 2. Materials and methods

This study explores the impact of participatory systems modeling on a youth mental health professional network in Bogota, Colombia. It was part of a broader program of research implemented in 2021–2022. The broader research program aimed to develop a system dynamics model that can be used as a decision-support tool for strategic planning and investments in youth mental health and suicide prevention in Bogota. The research program is a collaborative international endeavor between CSART (international), The University of Sydney (Australia), the Swiss Tropical and Public Health Institute (Switzerland), and Pontificia Universidad Javeriana (Colombia) in collaboration with a broad range of stakeholders in Bogota. It was anticipated that the modeling would inform policymaking on the selection of strategies and services needed to strengthen the youth mental health system and mitigate the social and economic drivers of youth mental health outcomes, e.g., the prevalence of youth mental health problems, emergency department presentations, and youth suicide, in Bogota, including the exacerbation of these outcomes due to the COVID-19 pandemic.

### 2.1. Approach

The project employed a participatory approach in the development of the system dynamics model following a similar participatory modeling approach conducted in Australia by the Brain and Mind Centre, University of Sydney^1^ (2). As in Australia, a collaboration between researchers, health policymakers, clinicians, and community actors in the local mental health system engaged in a participatory process to define system components, pathways, barriers, and the most critical interventions that could make an impact on suicidality, and overall mental health and wellbeing in Bogota’s youth.

A participatory process in system modeling includes actively involving stakeholders, such as experts, decision-makers, and affected community members, in various stages of the modeling process. It seeks to include diverse perspectives and knowledge to ensure a comprehensive and collaborative approach to understanding and addressing complex systems. A broad overview of the participatory system modeling process is as follows:

- Stakeholder Engagement: Stakeholders are identified and invited to participate in the modeling process. They may include experts from different domains, policymakers, community representatives, and other relevant parties.
- Problem Definition: Stakeholders work together to define the problem or issue that the system modeling aims to address. This ensures that the modeling effort is aligned with the concerns and priorities of all involved parties.
- Data Collection and Validation: Stakeholders contribute their expertise and knowledge to collect and validate data used in the modeling process. Their insights help ensure that the data accurately represents the real-world system being modeled. Both qualitative (interviews and surveys) and quantitative (surveys, existing datasets, etc.) data are collected. Stakeholders review the data in a validation workshop to ensure accuracy.
- Model Co-creation: Stakeholders collaborate with modelers to develop the system model. They actively contribute their insights, assumptions, and feedback throughout the modeling process in an iterative process.
- Scenario Development: Participating stakeholders are involved in generating and exploring different scenarios within the model. These scenarios can represent potential interventions, policy changes, or other strategies to address the identified problem.
- Model Interpretation and Policy Insights: Stakeholders engage in interpreting model results and drawing policy insights guided by the facilitators of the workshop. Their diverse perspectives can lead to a deeper understanding of the system’s behavior and the potential impacts of different interventions.
- Decision-making and Implementation: Participatory modeling aims to inform decision-making processes. Policymakers and stakeholders use the model results and insights to make informed choices and implement effective strategies.

By involving stakeholders throughout the system modeling process, participatory modeling fosters transparency, ownership, and collective learning. It enhances the credibility and relevance of the model’s outcomes and ensures that the modeling effort serves the interests and needs of the people affected by the system under study. This is achieved by providing a safe environment where participants are able to contribute without been judged, there are no right or wrong answers, they hear the participants’ opinions and as such they can also reflect on the mental health network and the relevance of them in their work. In addition, by hearing each other concerns, expertise, contribution and efforts, participants also got a basis to reflect in the importance of communication and collaborative work.

### 2.2. Evaluating networks in participatory systems modeling

The participatory process was conducted with a multidisciplinary group of stakeholders through three workshops. The participants were purposefully selected from the local Bogota youth mental health system and included a diversity of policymakers, providers at all levels of care, community representatives, representatives across broader health and social systems (e.g., education sector, health sector), special interest groups, civil society, young people with lived experience of mental illness, and caregivers of young people with lived experience. The SNA was part of a broader multi-scale and comprehensive evaluation approach that was employed in Bogota following a similar evaluation process conducted in Australia (12, 13). The results of the broader evaluation will be reported elsewhere, with this paper focused only on the SNA component as a complementary tool to provide insights that otherwise would be missed.

### 2.3. Social network analysis

We followed the stakeholders’ views and perceptions of their network, before, during, and after the series of modeling workshops to explore how the participatory process affected participants’ perspectives and collaboration/connection within the Bogota youth mental health network. Since we could not identify a validated survey to be applied to our needs, we followed the process suggested by Monaghan et al. (14) of using data from questionnaires and semi-structured interviews. Therefore, we used the information collected via an online survey and virtual interviews. Following Monaghan et al. (14), the data from the online survey was used to map the network and the linkages between the members of the network and the virtual interviews were used to understand the context of some of the answers of the online survey. We aimed to understand the linkages between stakeholder services and interests, their current relationships, and ways of working before and after the series of workshops. We were interested in potential changes in stakeholders’ perceptions about their existing network dynamics and relationships, e.g., changing their perceptions of potential partnerships and collaborations with other members of the network to improving mental health care for young people in Bogota.

To determine the impact of the PSM on the local social network of mental health, we used a basic SNA, which is a quantitative method that uses graph theory and sociograms to analyze and visualize social relationships, where nodes represent the actors, and lines represent their relationships (15). Therefore, we examined workshop participants’ social networks and the effects that participating in the workshops had on these networks, specifically on existing relationships. The SNA was performed to (*i*) map the stakeholders involved, the way they are linked, assess the influence of the actors, and determine the structure of the network; and (*ii*) how these former characteristics change after the participatory activities. All research activities were approved by the Institutional Ethics Committee of the Pontificia Universidad Javeriana (protocol number 2020/039) and participants provided their informed consent to take part in the study.

### 2.4. Participant sampling

Stakeholders were recruited to attend three participatory modeling workshops between the September 2021 and March 2022, which coincided with the COVID-19 pandemic. This imposed several restrictions that required adaption of the usual conduct of workshops. For example, each workshop was undertaken as several smaller workshops over the course of a week rather than as one large workshop on a single day—due to social distancing requirements and constraints on room capacity—which might or might not have restricted the ability of all stakeholders to engage with each other. Purposive sampling was used to identify and recruit participants for the SNA within the range of expertise needed. The participants selected from Bogota were representative of the diversity of stakeholders in the system.

### 2.5. Data collection and analysis

Shortly after recruitment, the participants were interviewed using a semi-structured interview guide to capture initial impressions and perceptions before their engagement in the participatory process. They were also requested to complete a survey before the first workshop and following the third workshop. The survey questionnaire complemented the interviews by asking similar questions to validate the qualitative data; but the survey, however, contained additional question modules to collect social network data from the stakeholders before and after the participatory workshops. The survey was created using the REDCap software and distributed using a digital link sent by email or instant messaging apps to all the participants before the first workshop (baseline), and after the third and final workshop (end line). The respondents could fill out the survey either in advance of their participation or at the start/end of the workshop. The surveys and interviews contained several questions that served the broader evaluation and are reported somewhere else. The survey’s specific module on SNA contained questions to find out to whom each of the represented organizations was connected, what kind of professional relationship or link they may have, i.e., administrative, implementation or research, and the frequency of the interaction between the linked organizations (see Table 1).

**Table 1:** Questions in survey related to SNA.

Does your organization collaborate with the following stakeholders because it is obliged to do so (e.g., because it is required to do so by a protocol, law, regulation)? Options: list of stakeholders’ category plus yes, no, or no relation.
Please indicate how close you are, in the work you do for your organization, with the following institutions. Consider issues of confidentiality, reliability, among others. Options: list of stakeholders’ category plus scale 0 (no relation) to 5 (very close)
Please evaluate the type of relationship your organization has with the following actors. Administrative/legal relationship: For example, one party imposes monitoring and control mechanisms, performance criteria and/or requests information; and the other party complies with them, renders accounts/reports and/or must request authorizations for its actions. Implementation relationship: For example, the organizations work together on programs, events or campaigns. Research relationship: For example, organizations collaborate to carry out knowledge production processes. Options: list of stakeholders’ category plus administrative, implementation, legal, or non-applicable.
Please evaluate the frequency with which your organization interacts with the following stakeholders. Options: list of stakeholders’ category plus regularly, occasional, rarely, or non-applicable.

The data collected through the surveys before the first workshop and after the third workshop was analyzed to detect changes in the connections or structural patterns measured. Only those participants that attended all three workshops were considered in the analysis to reduce the risk of confounders and increase the attribution likelihood to the workshop (16).

A network was defined as a number of nodes that are connected by interrelations called links. In SNA, the nodes are people or institutions and the links are any social connection between them. For this SNA, the nodes are persons and institutions represented at the workshops. We assessed relationships between nodes using different measures, the most basic being the number of contacts between two nodes, called “tie strength” (17). We also measured the key nodes—“network centrality”—using different aspects of centrality (18). For example, the nodes that receive the most links (*indegree centrality*), i.e., the nodes with whom most of the other nodes have an interaction with, the nodes that send the most links (*outdegree centrality*), i.e., the nodes that have the most interactions with other nodes, and the distance between nodes measured by their links (*degree centrality*) (19). We also assessed the approximate importance of each node using a measure called *Eigenvector*, which looks at the number of links a node has to other nodes in the network and how well connected those other nodes are through the network (*Eigenvector centrality*) (20). The complete set of used measures is explained in Table 2.

**Table 2:** Metrics used for the social network analysis.

Metric	Description
Degree	Degree centrality is the simplest of the centrality metrics, counting the number of connections an element has. In general, elements with high *degree* are the local connectors/hubs, but aren’t necessarily the best connected to the wider network.
Closeness centrality	Closeness measures the distance each element is from all other elements. In general, elements with high closeness can spread information to the rest of the network most easily and usually have a good overview of what is happening across the network.
Betweenness centrality	Betweenness centrality measures how many times an element lies on the shortest path between two other elements. In general, elements with high betweenness have more control over the flow of information and act as key bridges within the network. They can also be potential single points of failure.
Size	Size measures the number of neighbors an element has (plus the element itself). It’s similar to degree, but counts the number of elements instead of connections.
Indegree	Indegree measures the number of incoming connections for an element. In general, elements with high Indegree are leaders, looked to by others as a source of advice, expertise, or information.
Outdegree	Outdegree measures the number of outgoing connections for an element. In general, elements with high outdegree can reach a high number of elements and spark the flow of information across a network (but may not be the most efficient at spreading the information).
Eigenvector	Like other centrality measures, Eigenvector measures how well connected an element is to other well connected elements. That is, it measures a node’s influence based on the number of links it has to other nodes in the network, but also taking into account how well connected that node is, and how many links their connections have. In general, elements with high eigenvector centrality are the leaders of the network, though they may not have the strongest local influence.
Reach (two-step out)	Reach measures the portion of the network within two steps of an element. In general, elements with high reach can spread information through the network through close friend-of-a-friend contacts.
Reach efficiency	Reach efficiency normalizes reach by dividing it by size (number of neighbors). In general, elements with high reach efficiency are less connected but gain more exposure through each direct relationship.
MICMAC	MICMAC is a system analysis that explores element exposure (how much a given element is affected by other elements) and influence (how much a given element affects other elements). When plotted on an XY axis, these scores help you identify potential leverage points within the overall system.
Density	How many links between nodes exist compared to how many links between nodes are possible
Reciprocity	The likelihood of nodes in the network to be mutually linked
Diameter	The length of the longest path between two nodes
Avg. degree	The average number of links per node in the network
Avg. path length	The average number of steps along the shortest paths for all possible pairs of network nodes

In the survey, we asked the participants to identify the group that they were representing and to indicate the relationship that their organization has with other stakeholders within the network. As shown in Table 3, we also asked them to grade the “closeness” of their contact or relationship to the other stakeholders with whom they maintain a link, the type of link (e.g., research, implementation, or legal), and the frequency of their contact (very often to rarely). As other studies focused on SNA impacts (21–25), we analyzed the survey data using Kumu (Jeff and Ryan Mohr, web-based open source, version 2022 available at https://kumu.io). Kumu is an online system mapping tool and visualization platform for mapping systems and relationships that facilitates quantitative analysis using the metrics shown in Table 2.

**Table 3:** Workshop stakeholders’ categories, broken down according to youth mental health sectors engaged.

Categories of stakeholders represented in the workshops	Number of institutions represented during the 3 workshops (*n* = 32)
Policy Makers, Health Department, and Local Health District representatives	4
Youth Mental Health Academics, Social Scientists and Epidemiologists	4
Mental Health Clinicians	4
Health providers	5
Insurance reps/Health Benefit Plan Management Companies	1
Primary Care, GPs, and Allied Health Professionals	1
Child Protection representatives	2
Consumers/People with lived experience/carers	2
Education sector representatives (e.g., counselors, Education Department)	4
Non-Governmental Organizations (NGOs)	1
Representatives from special interest groups	4

## 3. Results

A total of 78 stakeholders were recruited to attend three participatory modeling workshops. The initial workshop was attended by 57 participants; the second workshop by 42; and the third workshop by 54. However, for this analysis, only the participants that attended all three workshops and answered both pre and post surveys were considered in the analysis (*n* = 24). The considered participants represented 11 stakeholder group categories (see Table 3) and 32 nodes were identified. This discrepancy between nodes and number of participants is due to several participants representing more than one institution. The list of the nodes and the groups they represent are in Table 3. A full list of the stakeholder categories is provided in Table 4.

**Table 4:** 32 nodes and the groups they represent in the network.

Node name	Group representing
Centre for Research on Youth Mental Health	Mental Health Clinicians
Children and Adolescent Protection Institute	Child Protection representatives
Colombian Association of Bipolars	Young people, consumers, people with lived experience, and carers
Colombian Association of People with Schizophrenia and their Families	Young people, consumers, people with lived experience, and carers
Colombian Occupational Therapy Association	Primary Care, GPs, and Allied Health Professionals
Colombian Psychiatric Association	Mental Health Clinicians
Corporación Nuevos Rumbos	Youth Mental Health Academics, Social Scientists and Epidemiologists
Education District Department	Education sector representatives (e.g., counselors, Education Department);
Emergency Services or Police	Policy Makers, Health Department, and Local Health District representatives
Emmanuel Clinic	Health providers
Foundation/NGO	NGO
Health District Department	Policy Makers, Health Department, and Local Health District representatives
Hermanas Hospitalarias del Sagrado Corazón de Jesús	Health providers
Insurance representatives/Health Benefit Plan Management Companies	Insurance companies
Javesalud	Health providers
Ministry of Health	Policy Makers, Health Department, and Local Health District representatives
National Health Observatory	Youth Mental Health Academics, Social Scientists and Epidemiologists
Online Provider of youth mental health services	Health providers
Organizations of people with lived experience/carers	Special interest groups representatives (e.g., armed conflict victims, LGBTIQ+)
Other District Department	Policy Makers, Health Department, and Local Health District representatives
Provider of youth mental health services	Health providers
Psychology Consultants, Pontificia Universidad Javeriana	Mental Health Clinicians
Public Health Institute, Pontificia Universidad Javeriana	Youth Mental Health Academics, Social Scientists and Epidemiologists
Public organization for the protection of children and adolescents	Child Protection representatives
School of Medicine, Pontificia Universidad Javeriana	Youth Mental Health Academics, Social Scientists and Epidemiologists
School of Psychology, Pontificia Universidad Javeriana	Mental Health Clinicians
Social or community organization	Special interest groups representatives (e.g., armed conflict victims, LGBTIQ+)
Spiritual or religious organization	Special interest groups representatives (e.g., armed conflict victims, LGBTIQ+)
Stonewall Student Group	Special interest groups representatives (e.g., armed conflict victims, LGBTIQ+)
Universidad de los Andes	Education sector representatives (e.g., counselors, Education Department)
University counseling and psychological services, Universidad de los Andes	Education sector representatives (e.g., counselors, Education Department)
University counseling and psychological services, Universidad Javeriana	Education sector representatives (e.g., counselors, Education Department)

**Figure 1:**
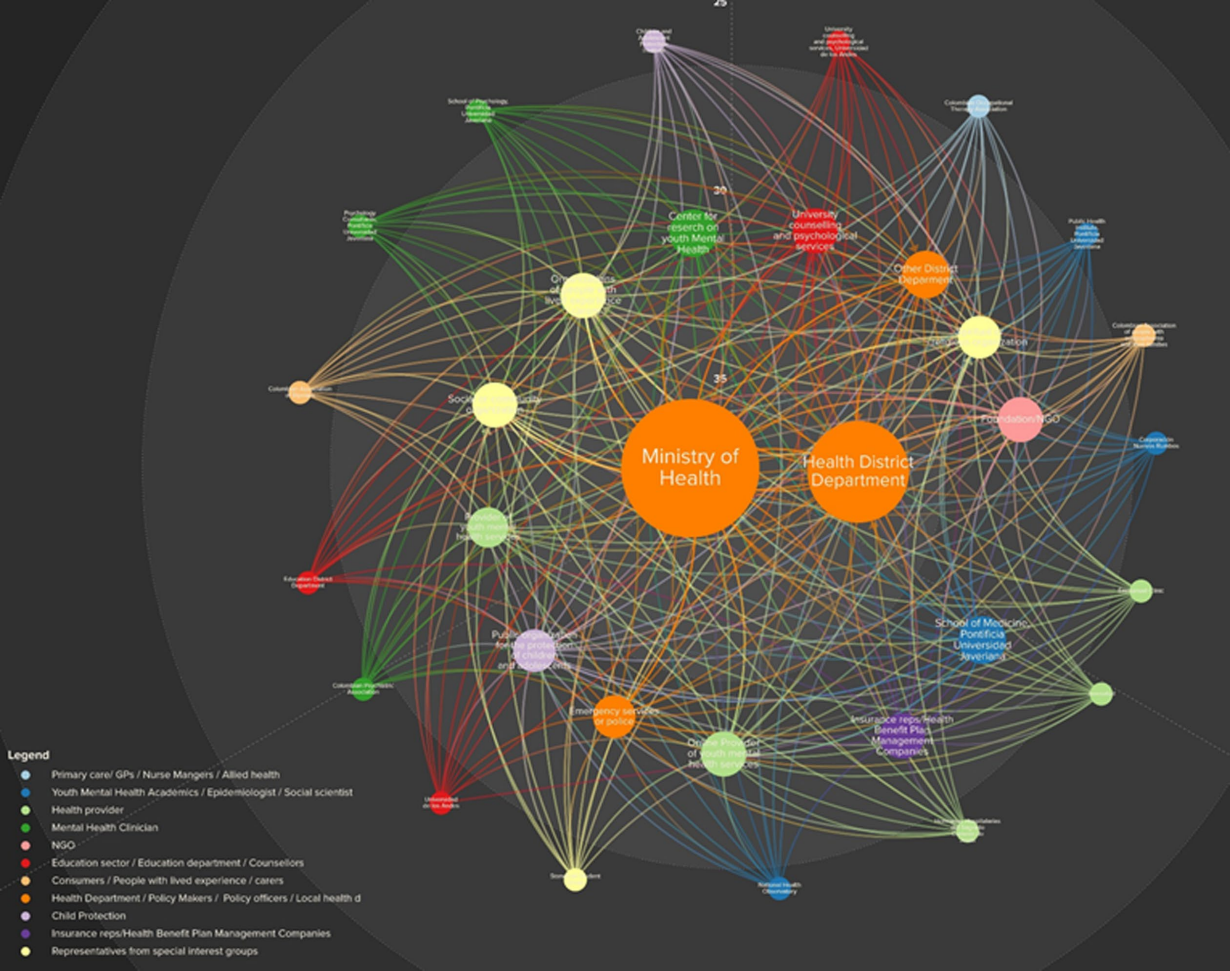
**(A)** Baseline social network structure for mental health in Bogota, Colombia. The circles represent stakeholders (nodes) and the links are the lines connecting two stakeholders. The node size is proportional to the node’s degree and the node color was coded according to the type of group they belong to. SNA measures: 32 elements, 321 connections, 0.64 density, 0.97 reciprocity, 3 diameter, 19.91 avg. degree, and 1.45 avg. path length. **(B)** End line social network structure for mental health in Bogota, Colombia. The circles represent stakeholders (nodes) and the links are the lines connecting two stakeholders. The node size is proportional to the node’s degree and the node color was coded according to the type of group they belong to. SNA measures: 32 elements, 308 connections, 0.57 density, 0.82 reciprocity, 3 diameter, 19.13 avg. degree, and 1.52 avg. path length.

We used the general network metrics (Table 2 and Supplementary Appendix) in both baseline and end line to describe the general characteristics of the network and created two graphic depictions based on these metrics, i.e., at a baseline and end line, shown in Figures 1A,B, correspondingly. For example, the node with the most connections indicates a main actor. In the same tenor, a node that is connected indirectly to most of the other nodes suggests an influential actor. By looking at these basic measures both before and after the workshops, we aimed to identify changes in the roles of the nodes if it was the case. These findings can help to describe a specific value obtained from the participatory process that may be overlooked by other evaluation methodologies. The values of the measurements of the whole network and their differences (baseline vs. end line) are shown in Table 5 showing no variation in the number of elements and the diameter of the network at baseline and end line suggesting a rigid network. Considerable changes were shown in the number of connections (decreasing from baseline to end line) and the average degree of the network (also decreasing from baseline to end line) suggesting changes in the role of some of the nodes.

**Table 5:** General network metrics.

Metrics	Baseline	End line	Difference
Elements	32	32	0
Connections	321	308	−13
Density	0.64	0.57	−0.07
Reciprocity	0.97	0.82	−0.15
Diameter	3	3	0
Avg. degree	19.91	19.13	−0.78
Avg. path length	1.45	1.52	0.07

Our SNA detected 2 main actors measured by the number of connections they have (degree centrality), namely the Bogota’s Health District Department (HDD) and the Ministry of Health and Social Protection (MoH), which did not change from baseline to end line (Figures 1A,B). Both organizations’ high centrality is a result of being identified by multiple respondents as the stakeholders with the most contacts. The circle size in Figures 1A,B is proportional to stakeholder centrality relative to the rest of the network and hence identifies the most connected stakeholders. According to our SNA, the network consists of only a single community, dominated by the HDD and the MoH based on their relative centrality value. This community did not change after the workshops.

In the dimension of closeness centrality, the MoH and the HDD remained as the dominating actors, before and after the workshops, both of which can easily spread information to the rest of the network and hence have high visibility. This holds also for betweenness centrality, denoting high control over the flow of information within the network from these two nodes. This aligns with the fact that both organizations also rank highest in outdegree (number of outgoing connections) and indegree (number of incoming connections) connections before and after the workshops. Both MoH and HDD are the leaders of the network (although not necessarily the only local influencers) according to their eigenvector centrality values (see Supplementary Appendix).

The reach efficiency measure shows the nodes less connected that gain exposure through each direct relationship. This measurement showed a change of nodes from the baseline to the end line (see Figures 1A,B and Supplementary Appendix). As per the MICMAC Cross Impact Analysis (26) in both exposure (how much a given element is affected by other elements) and influence (how much a given element affects other elements) the dominant actors also changed substantially as shown in Table 5. Regarding the size measure, i.e., the number of neighbors an element has plus the element itself, there were 12 changes from baseline to end line, and the reach measure, i.e., the portion of the network within two steps of an element, underwent seven changes. These measures are shown in Tables 5 6 and the remaining measures are shown in Supplementary Appendix.

**Table 6:** Stakeholders changes in MICMAC measures at baseline and end line.

Baseline		Endline	
Value	Stakeholder	Value	Stakeholder
MICMAC Exposure			
1	Ministry of Health	1	Universidad de los Andes
1	Health District Department	0.76	Ministry of Health
0.72	Children and Adolescent Protection Institute	0.68	Colombian Association of people with schizophrenia and their families
0.56	Center for research on youth Mental Health	0.68	Javesalud
0.56	Organizations of people with lived experience/carers	0.68	National Health Observatory

MICMAC influence			
Value	Stakeholder	Value	Stakeholder
1	Children and Adolescent Protection Institute	1	Ministry of Health
1	School of Medicine, Pontificia Universidad Javeriana	0.80	Social or community organization
1	Colombian Association of Bipolars	0.77	Health District Department
1	Colombian Occupational Therapy Association	0.67	Other District Department
1	Colombian Psychiatric Association	0.65	Insurance reps/Health Benefit Plan Management Companies

The MICMAC Exposure measures how much a given element is affected by other elements; whereas MICMAC influence measures how much a given element affects other elements. For both measures a higher value means higher affectation or higher degree of affectation. The value range is 0 (min) to 1 (max).

## 4. Discussion

Using a SNA to identify characteristics of a given network such as its most important actors, the most influential ones, and the visibility of others within the network, can be informative in understanding and leveraging social networks in the implementation of decisions made on the basis of the systems modeling tool. This study aimed to detect the impact of a participatory modeling approach on the youth mental health stakeholder network in Bogota, Colombia and contribute to the evidence on this topic. The findings suggest that the use of SNA can be an important tool to understand the change over time in a professional network and the role and influence of stakeholders within it after a PSM. For example, similar to a physical network, a social network has an intrinsic level of rigidity, which is the degree of resistance to change or deformation in its structure (27, 28). Rigidity level provides an overview of several aspects such as, e.g., how easily a network can be modified or how inflexible it can be. The level of rigidity can be influenced by factors such as the number of connections between nodes, the strength of those connections, and the diversity of nodes in the network (28).

Our findings demonstrated that in Bogota, the network’s general measures, such as the number of connections, density, reciprocity, and average degree diminished from the baseline to the end line indicating small changes within the network and the role its members play as a result of the participatory process. These findings suggest that indeed, the participatory process had an impact on the network.

The most important changes were:

Connections: The number of connections in the network decreased from 321 at baseline to 308 at endline. This indicates that some connections were lost or dissolved during the intervention period.

Density: The network density decreased from 0.64 at baseline to 0.57 at endline. The decrease in density suggests that the network became less connected or more fragmented over time.

Reciprocity: Reciprocity measures the proportion of mutual connections in the network. It decreased from 0.97 at baseline to 0.82 at endline. This indicates that fewer connections were mutual, and there was a decrease in the level of reciprocity in the network.

Diameter: The diameter remained constant at 3 for both baseline and endline, indicating that the overall reach of the network did not change significantly.

Average Degree: The average degree slightly decreased from 19.91 at baseline to 19.13 at endline. The average degree represents the average number of connections each node has in the network. The decrease in average degree suggests that, on average, each node had *slightly* fewer connections at the endline compared to baseline.

Average Path Length: The average path length increased from 1.45 at baseline to 1.52 at endline. The increase in average path length indicates that the network became *slightly* less efficient in terms of communication and information flow between nodes in relation to the network’s original structure.

Changes in MICMAC Exposure:

At baseline, the Ministry of Health and Health Department had the highest exposure values (both at 1), indicating that they were significantly affected by other nodes in the network.

At endline, the Universidad de los Andes and the Ministry of Health had the highest exposure values, suggesting a shift in the nodes that were most influenced or affected by others.

Changes in MICMAC Influence:

At baseline, several nodes had a high influence value of 1, including the National Health Observatory, School of Medicine, and others. This indicates that these nodes were influential in shaping the network’s dynamics.

At endline, the Ministry of Health, Social or community organization, Health District Department, and other entities emerged as influential nodes.

These findings impacted the network in the following ways:

Structural Changes: The decrease in the number of connections, density, and reciprocity, as well as the increase in average path length, suggest that the network became less cohesive and potentially less effective in information exchange and collaboration between nodes.

Shift in Influence: The changes in MICMAC influence reveal a shift in influential nodes within the network. At baseline, certain organizations, such as the National Health Observatory and School of Medicine, held significant influence. However, at endline, the Ministry of Health and other entities emerged as more influential, e.g., Insurance representatives (see Table 4).

Impact of the PSM: The participatory process likely led to changes in the network’s composition and dynamics. Some nodes may have gained or lost influence, resulting in alterations in the network’s structure and connections.

Role of Health Institutions: The high exposure values of the Ministry of Health and the Health Department at both baseline and endline indicate their crucial role as key nodes significantly affected by other entities in the network.

It may be that the most important stakeholders were consolidated during the workshops, causing less important stakeholders to “disappear” from the network, explaining the decrease in the connections and reciprocity in the whole network. However, other small yet important changes may have also taken place since the average path length in the network was a bit larger after the workshops, i.e., a “larger” path for all possible pairs of network nodes, suggesting a repositioning of certain stakeholders, moving further away.

For Bogota, the SNA measures not surprisingly showed the public health organizations as the most dominant within the mental health network, i.e., Ministry of Health and Health District Department, leading the number of connections and the flow of information. Indeed, as a policy-making institution, the MoH has a key influence in the mental health system. The measures that underwent the fewest changes between baseline and end line were reach and size, whereas the measures with the most changes were betweenness, eigenvector, and MICMAC influence.

Since reach and size are measures related to the structure of the network, the few changes may suggest that the network is quite rigid and not prone to change its structure easily. These findings also provide a hint that intersectoral collaboration may not be in place despite being currently encouraged by the local laws. However, the fact that measures related to influencing stakeholders presented so many changes before and after the workshops suggests that indeed, the workshops may have altered the perceived role of certain stakeholders. The participatory nature of the workshop may have facilitated contact and understanding of the multiple actors within the system, and thus changed the perception of some stakeholders within the “universe of actors” in the system. For example, according to the MICMAC influence, the Children and Adolescent Protection Institute was among the most influential stakeholders with a value of 1 at the baseline but was ranked among the least influential after the workshops, having an end value of 0. In contrast, the emergency services or police passed from a not influential value of 0 to one of the most influential ones, with a value of 0.96. Observations and discussions during the workshops about the role of emergency services may have changed the stakeholders’ perceptions of these services by the last workshop. We recognize it may be true that some of these results could be explained by the priming effects induced by the nature of the workshops, i.e., focused on youth mental health and participation (29). However, in this case this seems unlikely since there were also non-health related sectors represented in the workshops and other topics may have been more salient in some workshops, such as computational modeling.

Overall, the analysis suggests that the PSM had an impact on the social network’s structure, relationships, and dynamics. The shift in influential nodes and changes in connections and reciprocity indicate potential changes in the network’s effectiveness and ability to coordinate health-related activities. However, further investigation and qualitative analysis are required to understand the specific reasons behind these changes and the implications for the intervention’s objectives and outcomes at a larger term since, e.g., the possible loss in efficiency and cohesiveness captured by the SNA maybe a transitory phase toward a more efficient one.

In addition to the insights gained from the current study, SNA could offer a robust framework for future intervention development and implementation. The results of an SNA allows for targeted interventions by identifying key nodes or actors within the network who are most influential in specific aspects of a given intervention, thereby optimizing resource allocation. For example, by identifying the network member that can easily spread information an awareness campaign in mental health problems could be more effective if this member is involved in the design and implementation of the campaign. Moreover, SNA can be employed in the initial stages of intervention planning to conduct a comprehensive needs assessment, thereby ensuring that the intervention is tailored to the specific characteristics and needs of the network. The methodology also lends itself to the creation of an implementation map, pinpointing key stakeholders who are crucial at various stages of intervention rollout. Post-implementation, SNA can be a complementary tool for evaluating the impact of the intervention on network structure, offering quantitative metrics that can inform iterative improvements. Thus, SNA not only enhances our understanding of the current network dynamics but also provides actionable insights for the design, implementation, and evaluation of future interventions.

### 4.1. Limitations

A limitation of our study is that we sought to undertake only a basic SNA analysis leaving out some other aspects that could also have provided greater insights into how and why the participant network changed. This decision was made on the basis of not overburdening participants given their participation in the participatory modeling workshops as well as the broader evaluation with quantitative and qualitative components. However, the method used did answer the primary research question by being able to detect changes in the social network as a result of the participatory process. This demonstrates the value and feasibility of the approach as part of an evaluation framework for future applications of participatory systems modeling (13). Another limitation could have been the restrictions imposed as a result of the COVID-19 pandemic requiring the research team to divide each larger workshop into several sub-workshops. However, this could have had two possible and opposite consequences: *i*) Domination of groups of people preventing all the stakeholders from interacting with each other, or *ii*) increase of interaction due to being able to interact in smaller groups. Related to this, the compulsory wearing of masks may also have acted as a barrier to the development of interpersonal relationships that are important for networking. Finally, some participants attended the workshops on behalf of two or three different organizations, therefore, the answers to some questions from this type of participants could have been a mixture of perceptions of all the organizations that they represented and/or being a dominant perception by one of the organizations they represented.

## 5. Conclusion

Insights provided by this SNA suggest that the participatory approach to systems modeling can impact the relationships between stakeholders and organizations that provide mental health services to young people. The insights from an SNA can assist with identifying the ideal actors within the network, e.g., most influential ones, to distribute information, support initiatives, and foster collective action to improve the mental health system, which in turn can assist decision makers and program designers to form more efficient partnerships in the aftermath of a PSM. The information provided by an SNA can be combined with other insights in available from more traditional evaluation tools applied to participatory processes, e.g., qualitative insights, such as stakeholder interviews and observational notes, to provide additional context for network structures and relationships supporting the co-generation of local and scientific knowledge and cooperation between scientists and decision-makers.

## Data availability statement

The original contributions presented in the study are included in the article/Supplementary material, further inquiries can be directed to the corresponding author.

## Author contributions

SC: Conceptualization, Formal analysis, Methodology, Writing – original draft. AH: Writing – review & editing. LO-P: Writing – review & editing. MS-N: Investigation, Project administration, Writing – review & editing. DS-R: Data curation, Investigation, Validation, Writing – review & editing. GL: Writing – review & editing. J-AO: Funding acquisition, Investigation, Methodology, Project administration, Supervision, Validation, Writing – review & editing.

## Footnotes

^1^https://www.rightcarefirsttimewhereyoulive.com.au/
