# Supplementary material for: Can participatory processes lead to changes in the configuration of local mental health networks? A social network analysis
Source: Front Public Health. 2023 Nov 8;11:1282662. doi: 10.3389/fpubh.2023.1282662 (PMC10663236; doi:10.3389/fpubh.2023.1282662)
Supplement: Supplementary file 1 [file Table_1.docx]

**Appendix A. Results of the SNA measures at baseline and end line**
